# Locally rigid, vessel-based registration for laparoscopic liver surgery

**DOI:** 10.1007/s11548-015-1236-8

**Published:** 2015-06-20

**Authors:** Yi Song, Johannes Totz, Steve Thompson, Stian Johnsen, Dean Barratt, Crispin Schneider, Kurinchi Gurusamy, Brian Davidson, Sébastien Ourselin, David Hawkes, Matthew J. Clarkson

**Affiliations:** Centre For Medical Image Computing, Engineering Front Building, University College London, Malet Place, London, UK; Royal Free Campus, 9th Floor, Royal Free Hospital, UCL Medical School, Rowland Hill Street, London, UK

**Keywords:** Registration, Ultrasound, Laparoscopic, Liver surgery

## Abstract

**Purpose:**

Laparoscopic liver resection has significant advantages over open surgery due to less patient trauma and faster recovery times, yet is difficult for most lesions due to the restricted field of view and lack of haptic feedback. Image guidance provides a potential solution but is challenging in a soft deforming organ such as the liver. In this paper, we therefore propose a laparoscopic ultrasound (LUS) image guidance system and study the feasibility of a locally rigid registration for laparoscopic liver surgery.

**Methods:**

We developed a real-time segmentation method to extract vessel centre points from calibrated, freehand, electromagnetically tracked, 2D LUS images. Using landmark-based initial registration and an optional iterative closest point (ICP) point-to-line registration, a vessel centre-line model extracted from preoperative computed tomography (CT) is registered to the ultrasound data during surgery.

**Results:**

Using the locally rigid ICP method, the RMS residual error when registering to a phantom was 0.7 mm, and the mean target registration error (TRE) for two in vivo porcine studies was 3.58 and 2.99 mm, respectively. Using the locally rigid landmark-based registration method gave a mean TRE of 4.23 mm using vessel centre lines derived from CT scans taken with pneumoperitoneum and 6.57 mm without pneumoperitoneum.

**Conclusion:**

In this paper we propose a practical image-guided surgery system based on locally rigid registration of a CT-derived model to vascular structures located with LUS. In a physical phantom and during porcine laparoscopic liver resection, we demonstrate accuracy of target location commensurate with surgical requirements. We conclude that locally rigid registration could be sufficient for practically useful image guidance in the near future.

## Introduction

In the UK, approximately 1800 liver resections are performed annually for primary or metastatic cancer. Liver cancer is a major global health problem, and 150,000 patients per year could benefit from liver resection. Currently, approximately 10 % of patients are considered suitable for laparoscopic liver resection, mainly those with small cancers on the periphery of the liver. Potentially, laparoscopic resection has significant benefits in reduced pain and cost savings due to shorter hospital stays [7]. Larger lesions and those close to major vascular/biliary structures are generally considered high risk for the laparoscopic approach mainly due to the restricted field of view and lack of haptic feedback.

We have developed a system that provides wider spatial context and potentially greater accuracy by aligning a preoperative plan derived from magnetic resonance (MR) or CT scans with the laparoscopic view. In this paper, we describe a freehand laparoscopic ultrasound (LUS)-based system that registers liver vessels in ultrasound (US) with MR/CT data. Specifically, we evaluate whether within a small region of interest a locally rigid registration is sufficiently accurate for surgical guidance.

### Background

Previously reported commercial systems register using either surfaces of the liver reconstructed using a dragged pointer [14] or manual identification of four points (CAS-ONE^1^). The former will lead to errors due to direct contact with a soft tissue, while both are limited to a global rigid registration which is clearly unrealistic with the abdominal insufflation needed in laparoscopy. We have previously developed a system [24] for laparoscopic guidance based on dense stereo surface reconstruction [25] and an iterative closest point (ICP) [5]-based alignment to a surface derived from a preoperative CT model. However, the research literature suggests that deformable registration is a necessity for image guidance [13, 23]. But, deformable models are difficult to validate [19] and may have multiple plausible solutions. It is also essential that a surgeon understands the registration accuracy while operating. We therefore propose a system based on locally rigid registration and test whether such a system is sufficiently accurate for surgical guidance. In this paper, a LUS probe is used to scan a local region of interest and update the global rigid registration based on the alignment of vessels in the preoperative CT and intraoperative ultrasound data within the region of interest.

In the literature, Aylward proposed rigid body registration of 3D B-mode ultrasound to preoperative CT for radio frequency ablation, based on a feature-to-image metric [2]. Lange, however, used a feature-to-feature method by extracting vessel centre lines from CT and 3D power Doppler ultrasound and then used ICP followed by multi-level B-splines for non-rigid alignment [15]. This was subsequently extended to incorporate vessel branch points as registration constraints [16]. The branch points were automatically identified in advance of surgery in the CT data, but selected manually in the ultrasound.

Accurate segmentation is a critical prerequisite for feature-based registration, and ultrasound image segmentation is itself a challenging problem, due to the low signal-to-noise ratio. Noble provides a thorough review [20]. Subsequently, Guerrero used an ellipse model to constrain an edge detection algorithm [12], thereby extracting vessels from ultrasound data for assessment of deep vein thrombosis. Later, Schneider used power Doppler ultrasound to initialize and guide vessel segmentation in B-mode images [22], replacing the previously required [12] manual initialisation of vessel centres. A scale-space blob detection approach has been used by Dagon et al. [8] and Anderegg et al. [1] to initialise vessel regions and approximate vessel walls using an ellipse model.

An alternative approach to feature-to-feature registration is image-to-image registration. Penney et al. [21] transformed a sparse set of freehand ultrasound slices to probability maps and registered with resampled and preprocessed CT data. Subsequently, Wein et al. [26] used a magnetic tracker to perform freehand 3D ultrasound registration of a sweep of data to preprocessed CT, using a *semi-affine* (rotations, translations, 2 scaling, 1 skew) transformation. This work was extended to non-rigid deformation using B-splines and tested in a neurosurgical application [27].

Currently, there still exists challenges that are specific to the use of freehand LUS in surgical applications. The methods of Aylward et al. [2] and Lange et al. [16] are based on a 3D percutaneous probe. The probe is held stationary while a mechanical motor sweeps the ultrasound transducer in a predictable arc. Unfortunately, there are currently no commercially available laparoscopic 3D ultrasound probes. Wein’s work is based on a percutaneous probe, swept through a volume collecting a dense set of slices [26], and Penney’s work collects a sparse set of slices [21]. However, in a freehand laparoscopic setting, port positions and positioning of the LUS probe are often restrictive, and control of the motion during a sweep of data is often difficult, resulting in jerky motion. Moreover, the relatively small field of view makes the context difficult to interpret, and in our experience, it is often difficult to obtain elliptical vessel outlines.

### Contribution of this paper

In this paper, we describe a registration system to align models derived from preoperative MR/CT data to intraoperative freehand ultrasound data taken using a 2D LUS probe. The method has similarities to the preceding literature in that we extract vessel centre lines as in [1, 8, 22] and use an ICP registration as in [15, 22]. In addition, to the best of our knowledge, while globally rigid [22] and additionally deformable [1, 8] registration of vessel models from CT and US data have been proposed, an evaluation of registration accuracy in vivo in a clinically usable laparoscopic ultrasound system has not been reported. The hypothesis of this paper is that local rigid registration within a small region of interest is sufficient for image guidance without deformable modelling, and the specific contributions of this paper are the delivery of a system to achieve that, and a thorough evaluation of errors using a phantom and during porcine laparoscopic liver resection.

## Methods

**Fig. 1:**
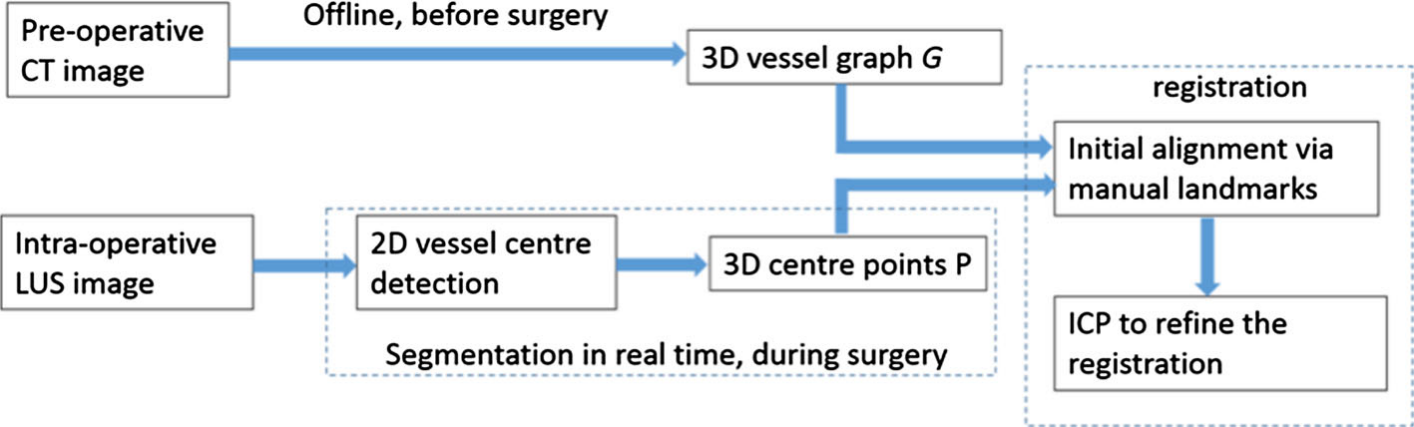
Overview of the registration process. Vessel centre points *P* from ultrasound data are registered to a vessel centre-line graph *G* giving rigid body transformation $^GT_{P}$

**Fig. 2:**
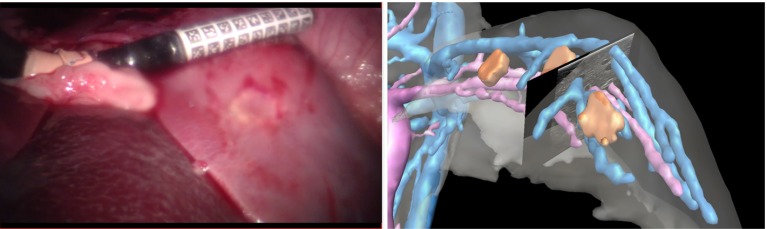
Applying the registration transformation to anatomical models derived from preoperative CT data enables live visualisation of CT data, within the context of live laparoscopic video and ultrasound data

Figure 1 shows an overview of the registration process. Vessel centre points *P* are detected in 2D ultrasound images and converted into 3D space via the ultrasound calibration and tracking transformations. The preoperative CT scan is preprocessed to extract a graph *G* representing vessel centre lines. The ultrasound- derived data *P* and CT-derived data *G* are registered using manually picked landmarks and/or the ICP algorithm. The rigid body registration transformation $^GT_{P}$ enables the preoperative data to be visualised relative to the live ultrasound imaging plane, as shown in Fig. 2.

### Preprocessing preoperative data

A standard clinical tri-phase abdominal CT scan is obtained and segmented^2^ to represent important structures such as the liver, tumours, arteries, hepatic vein, portal vein, gall bladder. Centre lines are extracted using the Vascular Modelling Tool Kit (VMTK).^3^ This yields a vessel graph *G*, which can be trivially processed to identify vessel bifurcation points.

### Real-time Ultrasound Segmentation

**Fig. 3:**
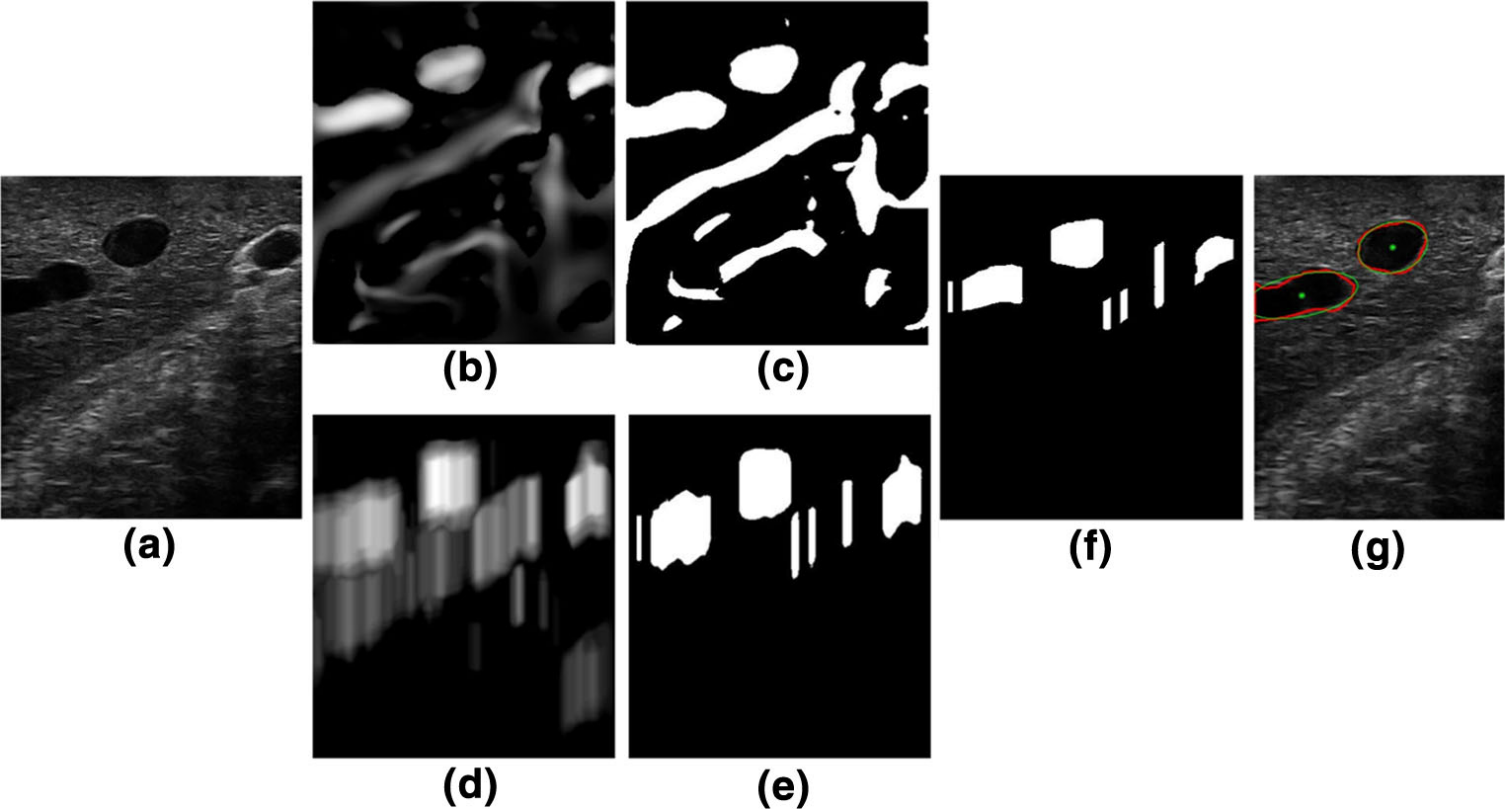
Vessel segmentation: **a** ultrasound B-mode image. **b** Vessel-enhanced image. **c** Thresholded vessel-enhanced image. **d** *Dip* image [21]. **e** Thresholded *Dip* image. **f** The candidate seeds of vessels after thresholded vessel- enhance image is masked with the thresholded *Dip* image. **g** Vessel contours are depicted in *red*, fitted ellipses, and centres are in *green*

Previous works on 2D ultrasound vessel segmentation use an ellipse model to constrain the edge detection process [1, 8, 12]. This approach assumes that vessels are imaged approximately perpendicular to the vessel centre line, which is not practical for laparoscopic use where movement is often restricted by the position of a trocar. Moreover, it is not clear how this approach handles topological changes of the external contours of vessels in the 2D US images. Therefore, we propose a flexible segmentation method that is not limited to cross-sectional scans and can cope with topology changes during the course of scanning. An example is shown in Fig. 3.

#### Vessel enhancement image

The standard B-mode ultrasound images have a low signal-to-noise ratio (Fig. 3a), so vessel structures are first enhanced for more reliable vessel segmentation. The multi-scale vessel enhancement filter [10] is used, which is based on eigenvalue analysis of the Hessian. The eigen values are ordered as $| \lambda _1 | < | \lambda _2 |$. The 2D “vesselness” of a pixel is measured by

1 $$\begin{aligned} v_0(s) = {\left\{ \begin{array}{ll} 0 &{} \text {if } \lambda _2 < 0 \\ \hbox {e}^{\left( - \frac{{R_B}^2}{2\beta ^2} \right) } \left( 1 - \hbox {e}^{\left( - \frac{S^2}{2c^2}\right) } \right) &{} \end{array}\right. } \end{aligned}$$

where

2 $$\begin{aligned}&R_B = \lambda _1 / \lambda _2 \end{aligned}$$

3 $$\begin{aligned}&S = \sqrt{{\lambda _1}^2 + {\lambda _2}^2} \end{aligned}$$

$\beta = 1$ and $c = 10$ are thresholds which control the sensitivity of the line filter to the measures ${R_B}$ and *S*. In Fig. 3b, it can be seen that some common artefacts on the ultrasound images, e.g. shadows, are wrongly picked up by the enhancement filter. For many cases, using only the prior knowledge of the vessel intensity distributions is not sufficient to exclude those non-vessel regions. To improve robustness, we adopt the *Dip* image as proposed by Penny et al. [21].

#### Creation of the Dip image

The *Dip* image ($$I_{dip})$$ was originally designed to produce vessel probability maps via a training data set. In this paper, we only use the intensity differences (i.e. intensity dips) between regions of interest. The size of a region is determined by the diameter of vessels. No additional artefact removal step is required, except for a Gaussian filter over the US image. Since we currently target the left liver lobe for surgical guidance, we set the search range of vessel diameters from 9 to 3 mm (roughly equal to 100–40 pixels on the LUS image) as a porcine left lobe features relatively large vessels.

**Fig. 4:**
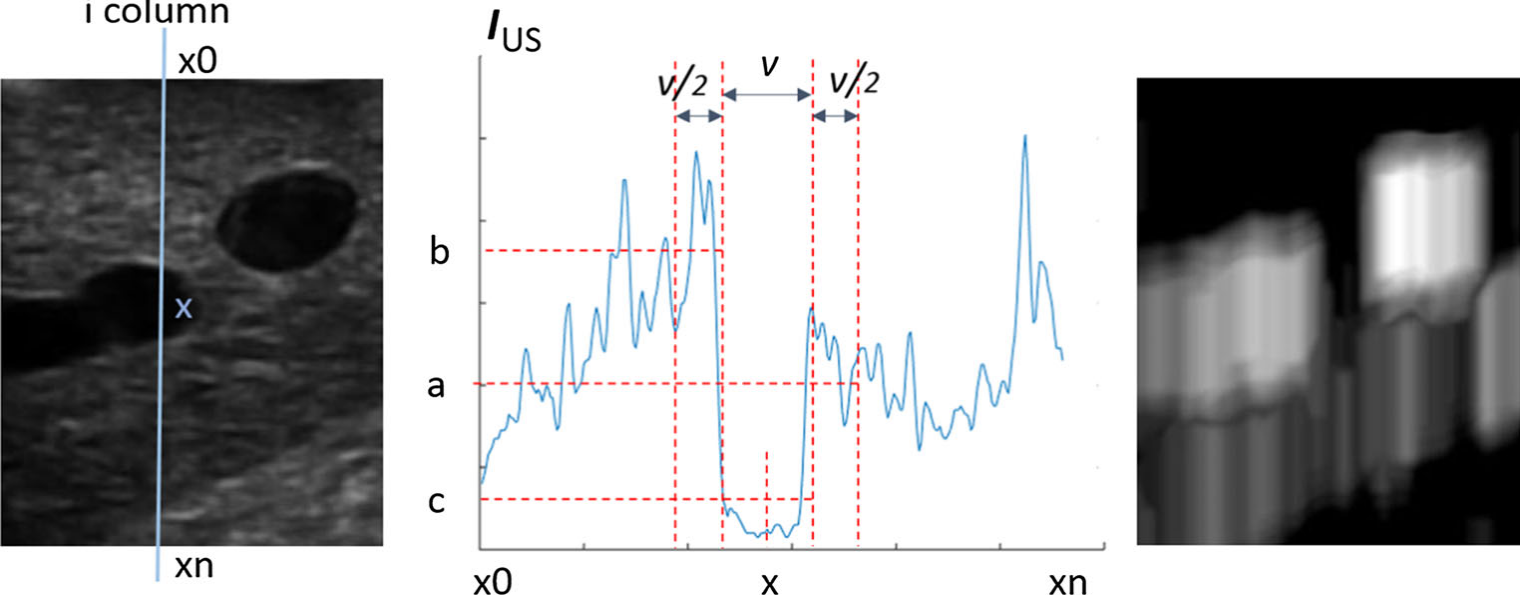
Creation of the Dip image. *Left* Gaussian blurred ultrasound image $(I_{US})$. *Centre* The intensity profile along line $(x_0, x_n)$. The location and size of image regions which gives the values *a*, *b* and *c*. *Right* The resulting *Dip* image

The *Dip* image is computed along the beam direction. As we use a linear LUS probe, the beam directions can be modelled as image columns. Figure 4 illustrates the calculation of three mean intensity values *a*, *b* and *c*, within regions $[x+v/2 , x+v], [x-v , x-v/2], [x-v/2 , x+v/2]$, respectively, with *x* being a pixel at the *i*th column and *v* the vessel width. If $c < b$ and $c < a$, every pixel in $[x-v/2, x+v/2]$ on the *Dip* image will have the value $b_v = \hbox {min} (a-c, b-c)$. This process is repeated for each v in $[v_\mathrm{min}, v_\mathrm{max}]$. The final pixel values at position $[x-v/2, x+v/2]$ will be $\hbox {max}(b_v)$. The steps above are repeated for every column of the US image and all pixels along that column. This can be parallelised easily as each column is processed independently of others. To reduce the search range of vessel diameters, a coarse-to-fine pyramidal approach is proposed to speed up the process further.

#### Segmentation and reconstruction

The vessel-enhanced image is thresholded at $T_\mathrm{e}$ to eliminate background noise; Fig. 3c. We create a mask image $(I_\mathrm{mask})$ by applying a threshold $(T_\mathrm{d})$ to the *Dip* image which is set as half of maximum value of the *Dip* image; Fig. 3e. These two thresholds are set for the given B-mode imaging parameters, e.g. gain, power, map.

The de-noised vessel-enhanced image is masked with $I_\mathrm{mask}$. Regions appearing on both images are kept (Fig. 3f). The intensity distribution of those regions will be further compared against the prior knowledge of vessel intensity and will be removed if they are not matching, i.e. falling out of the vessel intensity range. The remaining pixels are candidate vessel seeds. The regions in the de-noised vessel enhancement image containing such candidate seeds are identified as vessels and their contours are detected.

**Fig. 5:**
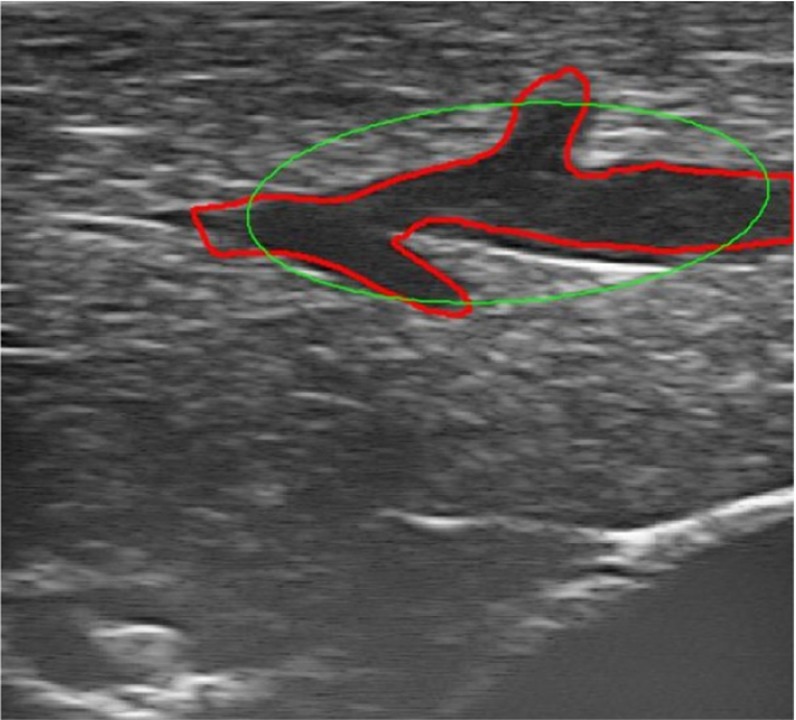
Example of outlier rejection. The ellipse is fitted to the vessel outline but the detected centre is rejected by the ellipse axes criteria

Since vessel centre points are employed for registration in this paper, ellipses are fitted to those contours to derive centre points in each ultrasound image (Fig. 3g). Outliers can be excluded by defining minimal and maximal ellipse axes ratio and length, as demonstrated in Fig. 5. For example, when an image is scanned near parallel to a vessel centre-line direction, it results in large ellipse axes. This can be removed by constraining the short axis length to the pre-defined vessel diameter range $[v_\mathrm{min}, v_\mathrm{max}]$, as described in section “Creation of the Dip image”. An additional criterion is that the axes ratio should be larger than 0.5. Otherwise, the vessel could be scanned less than 30$^\circ$ to its centre-line direction, which often does not produce reliable ellipse centres.

The vessel centres, in 2D pixel coordinates are multiplied by the ultrasound calibration, and the probe tracking transformation and hence converted into 3D data points (*P*), which are used to register the preoperative CT data to the patient in the operation room.

### Registration

**Fig. 6:**
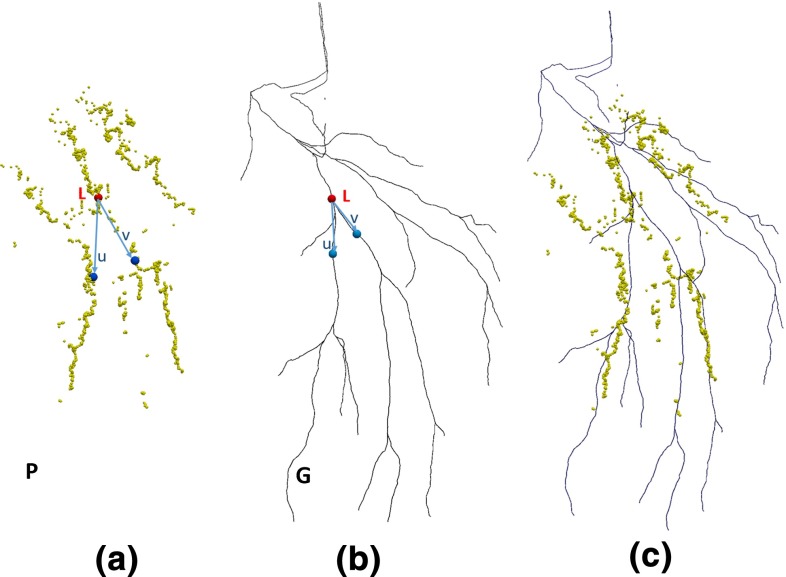
Example of corresponding landmarks and vectors in the hepatic vein, as used for initial alignment. **a** Intraoperative centre points *P*. **b** Preoperative centre-line model *G*. **c** Preoperative centre-line model *G* is aligned to intraoperative centre points *P* using ICP

Figure 6a, b illustrates the landmarks and vectors used for initial alignment. A landmark *L* and two vectors, $\mathbf{u}$ and $\mathbf{v}$, are defined on the preoperative centre-line model *G*, along with their correspondences $L', \mathbf{u', v'}$ in the derived centre points *P*. Currently, this was done manually. An initial rigid registration is obtained by the alignment of landmarks $\{L, L'\}$ which gives the translation, and vectors $\{\mathbf{u, u'}\}$ and $\{\mathbf{v, v'}\}$ which computes the rotation. After the initial alignment, the ICP algorithm [5] is applied to further refine the registration of preoperative data *G* to the intraoperative data *P* (Fig. 6c).

## Experiments and results

Experiments were performed to determine the overall registration accuracy of the system, and to identify sources of error from various component parts (sections “Ultrasound calibration error” and “Vessel segmentation error”). Our system uses an electromagnetic (EM) tracker, which is known to display tracking inaccuracies due to magnetic field inhomogeneities [11]. Various works have tried to mitigate against EM tracking inaccuracies by calibration [18] and combination with optical trackers [9]. The focus of this paper is the practicalities of intraoperative registration, so we refer to the manufacturer-claimed position accuracy of 1.4 mm RMS and orientation accuracy of 0.5$^{\circ }$ RMS. A fundamental point for surgical navigation is that while the presented algorithm determines the registration transformation $^{P}T_{G}$ from preoperative data *G* to intraoperative data *P*, the actual navigation accuracy will be the composition of the registration accuracy, the EM tracking accuracy as the probe moves, the US calibration accuracy and the deformation of the liver due to the US probe itself. For this reason, separate data are used to assess registration accuracy (section “Registration accuracy: in vivo”), and navigation accuracy (section “Navigation accuracy: in vivo”). In experiments “Registration accuracy: in vivo” and “Navigation accuracy: in vivo”, we use vessel models derived from CT scans taken using pneumoperitoneum (insufflated), which are not available clinically. So in section “Comparison of insufflated versus non-insufflated models”, we specifically compare registration and navigation accuracy when registering to CT-derived vessel models using pneumoperitoneum (insufflated) and without pneumoperitoneum (non-insufflated). US images were collected under controlled breathing (Boyles apparatus), which is discussed later.

### Experimental set-up

Our data acquisition system is built upon the NifTK platform [6]. Live LUS images are acquired at 25 frames per second (fps). We used an Analogic^4^ SonixMDP, and a Vermon^5^ LP7 linear probe. An Ascension^6^ 3D Guidance medSafe mid-range electromagnetic (EM) tracker was used to track the LUS probe at 60 fps via a six-degrees-of-freedom (6-DOF) sensor (Model 180) attached to the articulated tip.

### Ultrasound calibration error

**Fig. 7:**
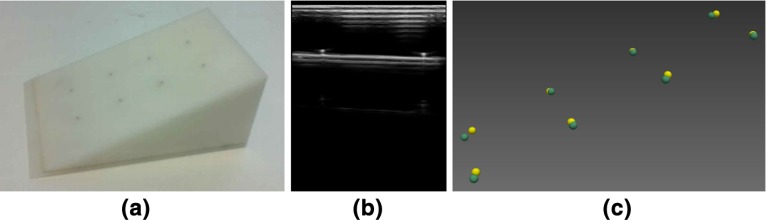
Evaluation of ultrasound calibration using an eight- point phantom. **a** Eight-point phantom. **b** A LUS B-mode scan of pins on phantom. **c** 3D positions of eight pins obtained from tracked LUS scans are depicted in *yellow*. The ground truth position of eight pins is depicted in *green*

The LUS probe was calibrated at a scanning depth of 45 mm before surgery using an invariant point method [17]. The scanning depth of the LUS probe was not changed throughout our experiments. The validation phantom is shown in Fig. 7a, and described further in [4]. Eight pins on the phantom were scanned in turn using the LUS probe. The pin heads were manually segmented from the US images, Fig. 7b. 100 frames were collected at each pin to minimise the impact of manual segmentation error. Their 3D positions in the EM coordinate system were computed by multiplying the 2D pixel location by the calibration transformation and then the EM tracking transformation, Fig. 7c. The accuracy of these computed 3D positions were validated based on two ground truths. The first ground truth is the known geometry of the 8-pin phantom, where the pins are arranged on a $4 \times 2$ grid, with each side being 25 mm in length. The resulting mean edge length was 24.62 mm. The second ground truth is the physical positions of the eight phantom pins in the EM coordinate system, which are measured by using another EM sensor tracked by the same EM transmitter, Fig. 7c. The distance between each reconstructed pin and its ground truth position is listed in Table 1.

**Table 1:** Error measures for each reconstructed pin position

	Pin number							
	1	2	3	4	5	6	7	8
RMS error (mm)	2.89	3.40	1.28	0.81	2.35	1.59	2.20	2.82

See Fig. 7c

### Vessel segmentation error

**Fig. 8:**
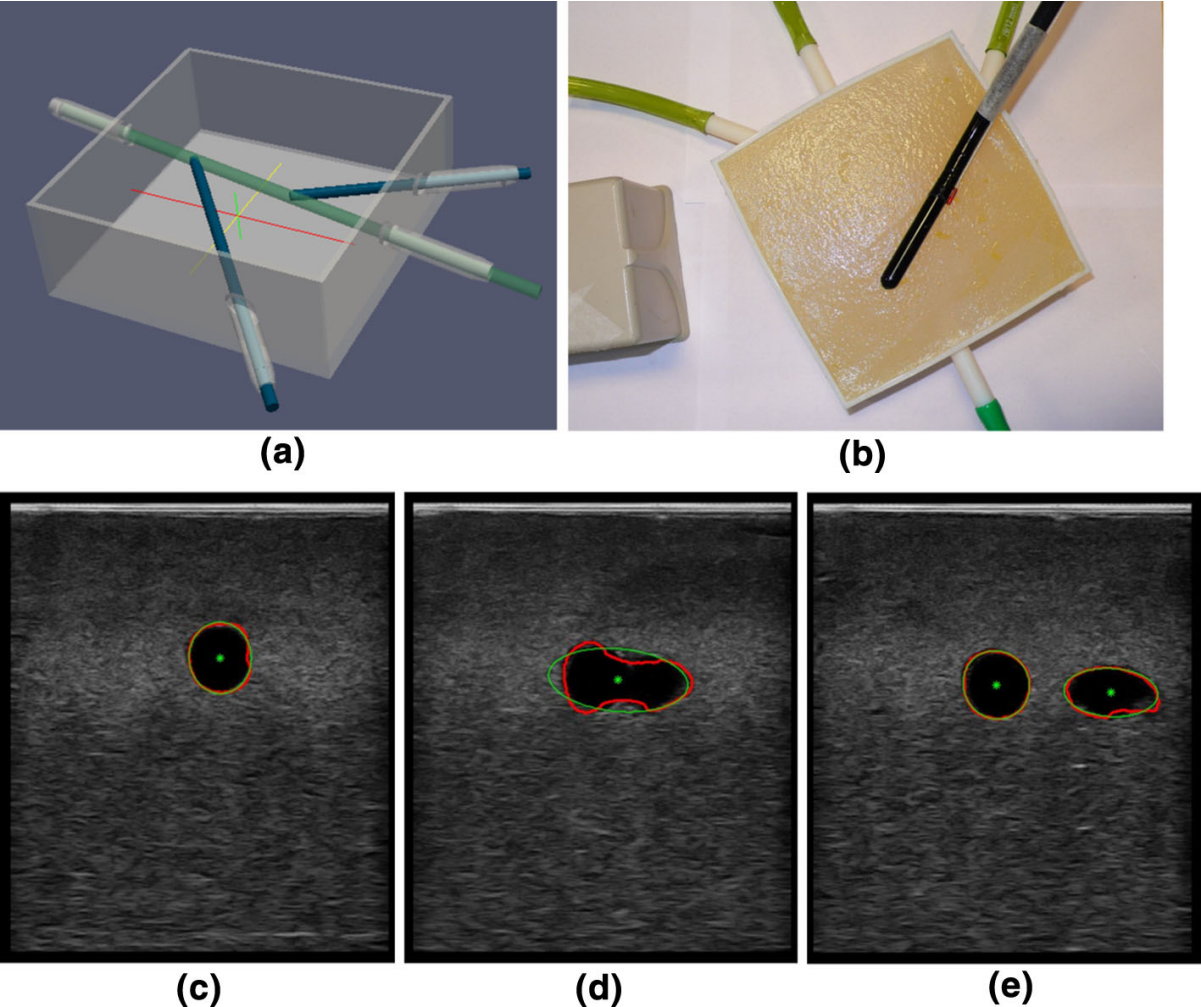
Validation of vessel segmentation on a phantom. **a** Phantom design. The rods will be removed after filling the *box* with Agar. **b** LUS probe sweeping across the surface of the phantom which is made from Agar. An EM sensor is attached to the probe and tracked. **c**–**e** LUS images of the tubular structures. The outlines are depicted in *red*. The ellipses fitted to the outlines are depicted in *green*. The extracted ellipse centres are depicted in *green*

LUS images were acquired from a phantom made from Agar. The phantom contained tubular structures filled with water, as shown in Fig. 8a, b. The ground truth is the diameter of the tubular structures manufactured as 6.5 mm. One hundred and sixty images ($640\times 480$ pixels) were collected. The contours of the tubular structures were automatically segmented from the US images and fitted with ellipses, so that the short ellipse axis approximated the diameter of the tubular structures, Fig. 8c–e. The resulting mean (standard deviation) diameter of the segmented contours was $6.4\;(0.17)$ mm. The average time of the image processing on one US image was 100 ms.

### Registration accuracy: phantom

**Fig. 9:**
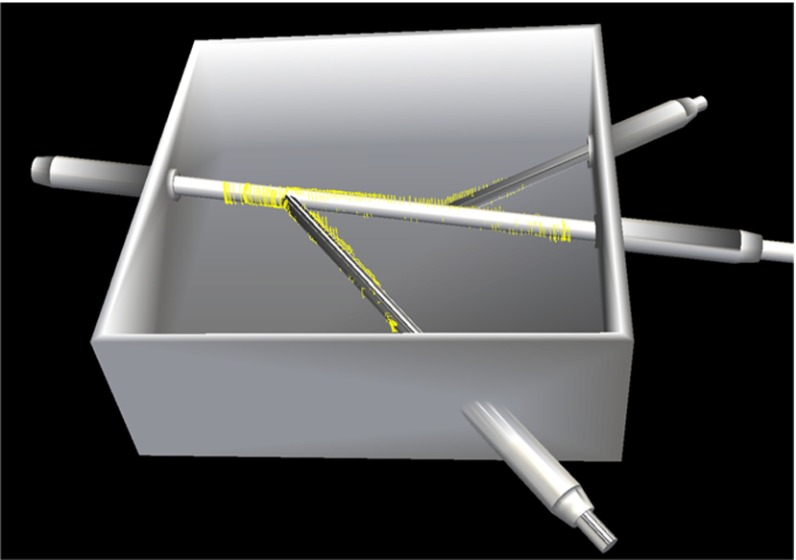
Validation of vessel registration on a phantom: the reconstructed contours from the ultrasound data (*yellow rings*) were rigidly registered to the phantom using ICP

The registration accuracy was assessed on the same phantom as section “Vessel segmentation error”, Fig. 8. Using the presented algorithm, the tubular structures were automatically segmented, the centre points extracted, and converted to EM coordinates by multiplication with the US calibration matrix and EM tracker matrix. These reconstructed points were rigidly registered to the centre lines of the phantom tubular structures using the ICP method. Figure 9 illustrates the registration of reconstructed points to the phantom model. The RMS residual error given by the ICP method was 0.7 mm.

### Registration accuracy: in vivo

**Fig. 10:**
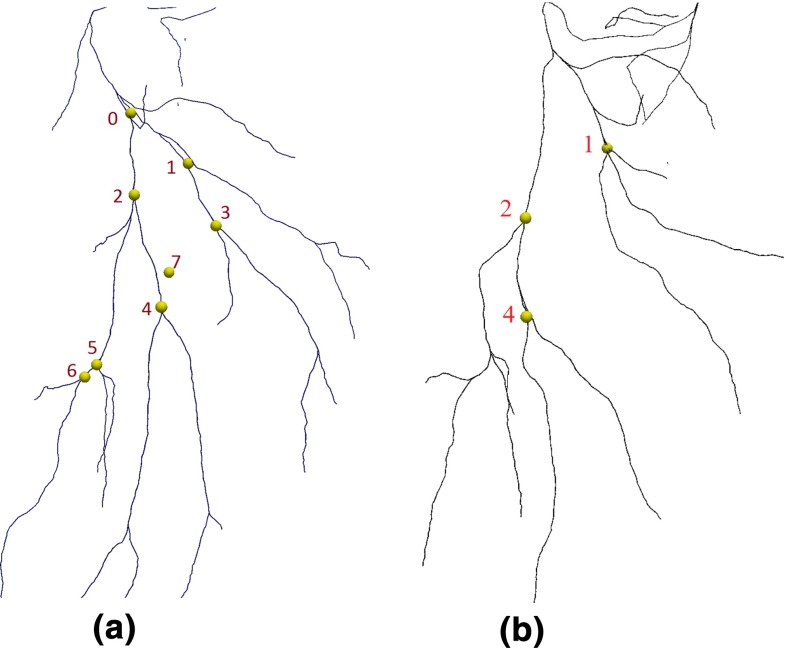
Hepatic vein landmark positions. **a** Eight bifurcation landmarks on the centre-line model were used to measure TRE in the first study. **b** Three bifurcation landmarks on the centre-line model were used to measure TRE in the second study

The overall registration accuracy was evaluated during porcine laparoscopic liver resection using two studies of the same subject. The LUS images were acquired from the left lobe of the liver, before and after a significant repositioning of the lobe. The surgeon swept the liver surface in a steady way to make sure vessel centre points were densely sampled and gently so as not to cause significant deformation of the liver surface. The US imaging parameters for brightness, contrast and gain control were preset values and not changed during scanning. About 10 images per second were segmented. In the first study, in total 370 images (640 $\times$ 480 pixels) were processed. In the second study, 340 images were processed. The detected vessel centres were converted into 3D data points *P*. Two tri-phase clinical CT scans had been obtained a week earlier, one with insufflation (12 mm Hg) and one without. Vessel centre lines were extracted using the model derived from the insufflated CT scan. The registration method of section “Registration” was applied, registering the preoperative centre-line model *G* to the intraoperative data set *P*. For the first study, eight bifurcations were manually identified and labelled in both the US images and the CT data, to be used for anatomical targets, as shown in Fig. 10b. The mean TRE was 3.58 mm, and the maximum TRE was 5.76 mm. For the second study, three bifurcations (i.e. number 1, 2, 4 in Fig. 10) were identified, as only the middle part of the left lobe of the liver was scanned. The mean TRE was 2.99 mm and the maximum TRE was 4.37 mm.

### Navigation accuracy: in vivo

To evaluate the navigation accuracy, the surgeon scanned another LUS image sequence for each study (giving four US data sets in total), again using minimal force on the LUS probe to avoid deformation. Using the same bifurcation landmarks as in the registration experiment (section “Registration accuracy: in vivo”), the corresponding landmarks on LUS images were manually identified. For the first study, the mean TRE was 4.48 mm and the maximum TRE was 7.18 mm. For the second study, the mean TRE was 3.71 mm and the maximum TRE was 4.40 mm.

### Comparison of insufflated versus non-insufflated models

**Fig. 11:**
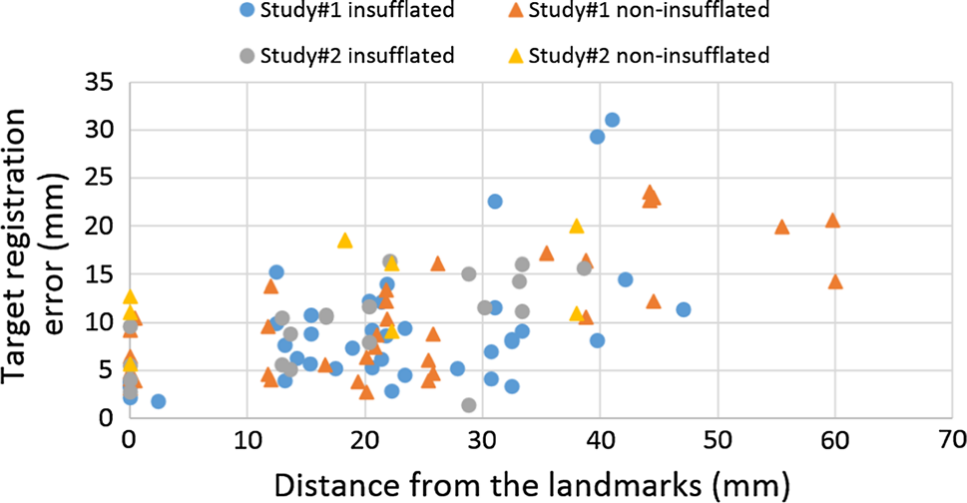
Evaluation of registration accuracy with locally rigid registration. The errors are shown as a function of distance from the landmark used to register. Within 35-mm distance to the reference points, 76 % landmarks have TRE smaller or equal to 10 mm with the insufflated CT model; 72 % for the non-insufflated CT model

**Fig. 12:**
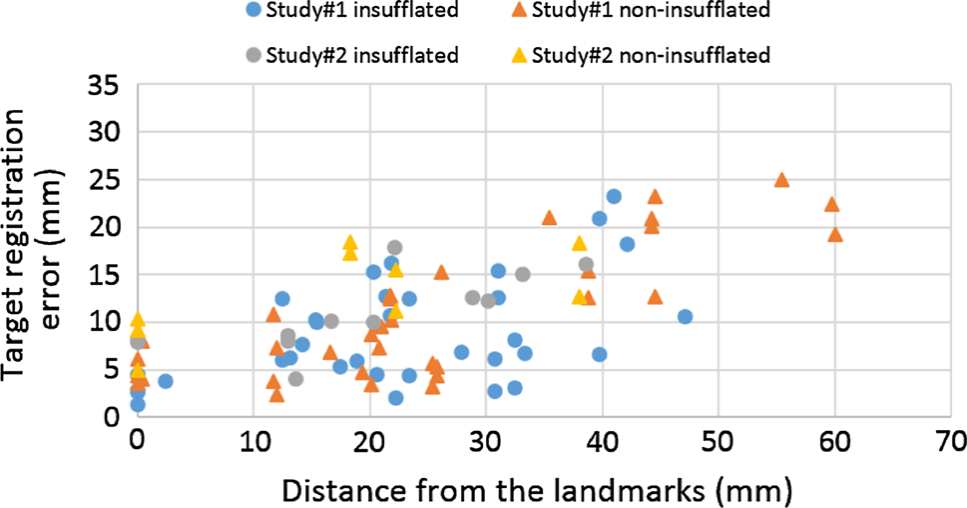
Evaluation of navigation accuracy with locally rigid registration. The errors are shown as a function of distance from the reference landmarks. Within 35-mm distance to the reference points, 74 % landmarks have TRE smaller or equal to 10 mm with the insufflated CT model; 71 % for the non-insufflated CT model

In sections “Registration accuracy: in vivo” and “Navigation accuracy: in vivo”, we used the insufflated CT model to evaluate the registration and navigation accuracy. In clinical practice, the patient would be scanned without insufflation, so in this section we used vessel centre lines derived from both insufflated and non-insufflated CT data. From the first study, landmarks 1, 2, 4, 5 (see Fig. 10a) were manually identified and labelled in both the US images and the CT data. From the second study, landmarks 1, 2, 4 (see Fig. 10b) were used. Using each landmark a registration was performed, registering the CT data to the US using the manual registration method (a landmark and two vectors, illustrated in Fig. 6a, b). For each registration the TRE was evaluated as in section “Registration accuracy: in vivo” using the eight bifurcations for the first study and the three bifurcations for the second study. The measures of TRE are presented graphically in Fig. 11. Similarly the navigation error is measured on the second LUS sequence for each study for each locally rigid registration. The measures of navigation error are illustrated in Fig. 12.

## Discussion

In this paper, we describe and evaluate a practical laparoscopic image guidance system based on a fast and accurate vessel centre-point reconstruction coupled with a locally rigid registration to the preoperative model using vascular features visible in LUS.

In section “Ultrasound calibration error”, we checked the accuracy of the invariant point calibration method. The mean edge length between pins in the 8-pin phantom was 24.62 mm compared with a manufactured edge length of 25 mm. Table 1 shows the reconstructed physical position errors between 0.81 and 3.40 mm, and an average of 2.17 mm, and this includes errors in measuring the gold standard itself. We concluded that although simple, this was comparable to other methods [17]. Future work could try improve calibration accuracy, specifically for LUS probes.

We subsequently checked the segmentation accuracy on a phantom (section “Vessel segmentation error”). The phantom was constructed via 3D printing a computer-aided design (CAD) model. So, the plastic phantom had known geometry with a tolerance of 0.1 mm. The reconstructed size of the internal diameter of the tubes was 6.4 mm compared with the diameter in the CAD model of 6.5 mm and was deemed within tolerance. Furthermore, in section “Registration accuracy: phantom” we see that the ICP-based registration of the point cloud resulting from the US segmentation to the CAD model itself gave a RMS error of 0.7 mm.

In section “Registration accuracy: in vivo”, we evaluated the registration accuracy in two in vivo studies. The mean TRE was 3.58 and 2.99 mm, measured at eight and three identifiable landmarks,respectively. This represents a best-case scenario for rigid registration, as we used an insufflated CT model, and a large region of interest (left temporal lobe). However, it does include movement due to respiration and cardiac pulsatile motion. The controlled breathing means that most of the time is spent near maximum exhale. We collected data for around 40 seconds, over several breathing cycles. Thus we assume that for the ICP-based methods, over a large region of interest, the data will be somewhat noisy, but the registration will average over the noise. For the manual landmark- based registration, future work will consider breath-holding techniques, faster software or a footswitch synchronised to the breathing. During the cardiac cycle, vessels pulsate and change size. We mitigated against this problem by using vessel centre lines which should be more reliable than vessel external contours.

From the initial registration, a second test data set was used to evaluate navigation accuracy. This incorporates the error due to registration, additional nonlinear EM tracking errors and errors due to further liver deformation via the US probe. Comparing the TRE errors of the corresponding data set in sections “Registration accuracy: in vivo” and “Navigation accuracy: in vivo”, the navigation accuracy is only slightly worse than the registration accuracy, if the surgeon performed the US scans in a consistent way. This also suggests the EM tracking error may not be a major problem, although further work is needed here.

In clinical practice, the patient will not be CT scanned while insufflated. The preoperative, non-insufflated CT will have a significantly different shape to that seen during surgery. So we compared registration of both insufflated and non-insufflated CT. It was difficult to identify corresponding landmarks in both CT scans. So rather than having eight landmarks in study 1, we could only identify landmarks labelled as 1, 2, 4 and 5 in Fig. 10a consistently in both insufflated and non-insufflated CT models. If a large region of interest was scanned using the US probe, the ICP-based registration to non-insufflated CT models was unreliable, due to the significantly different shape. If a small region of interest was scanned, then the smaller the structure, the more likely it was to be featureless, e.g. more closely resembling a line. So, to directly compare insufflated with non-insufflated registration, the manual landmark- based method (section “Registration”) was used around individual bifurcations, so as to be consistent across the two studies. Comparing Figs. 11 and 12, we can see that there are similar errors when using non-insufflated or insufflated errors. But an acceptable level ($<$5 mm) is achievable only near to a registration point. Interestingly, the navigation errors are similar. We tested locally rigid registrations on both insufflated and non-insufflated CT models which gave mean (standard deviation) errors of 4.23 (2.18) mm and 6.57 (3.41) mm, when measured at target landmarks located within 10 mm of the landmark used to register. When measured within 35 mm to the reference points, over 70 % of the target landmarks have errors smaller or equal to 10 mm for both model. Figures 11 and 12 show that if TREs are assessed away from the reference points, then errors do indeed increase.

Considering the state of the art in finite element methods that do attempt to compensate for tissue deformation, Suwelack et al. [23] measured errors of 5.05 mm and 8.7 mm on a liver phantom, Haouchine et al. [13] measure registration accuracy at two points as 2.2 and 5.3, in an ex-vivo trial while Bano et al. [3] measure 4 mm error at the liver surface but 10 mm error at structures internal to the liver. Although deformable models based on understanding of the biomechanics of tissue deformation are developing rapidly [3, 13, 23], there remain significant issues of validation in a surgical environment. We anticipate that it will be a long time before the surgeon has sufficient faith in a deforming model alone to guide surgical decisions during resection itself. However we do propose that this locally rigid registration system is practical and could relatively easily be automated with minimal user intervention. Local regions could also be used to drive and validate a deformable model.

## Conclusion

While this work is preliminary and performed on two studies from a single porcine experiment, we believe that the results provide preliminary evidence that our method is sufficiently accurate to be further developed and validated using animal models and clinically. Given a simple interface and a sufficiently close initial estimate, the liver could be scanned round the target lesion and nearby vessel bifurcations, and then it may be possible to obtain registration errors of the order of 4–6 mm with no deformable modelling. Our proposed method is both practical and provides guidance to the surgical target. It also implicitly includes information on the location of nearby vasculature structures which are the same structures that the surgeon needs to be aware of when undertaking laparoscopic resection. It may be that such a system has advantages over open surgery and haptics where the surgeon still remains blind to the precise location of these structures. Future work, considering either the vessel centre lines and deformable registration or combining ultrasound vessel centre lines into a deformable model will likely provide either better accuracy, more robustness or both.

## Footnotes

1 http://www.cascination.com.

2 http://www.visiblepatient.com.

3 http://www.vmtk.org/tutorials/Centerlines.html.

4 http://www.analogicultrasound.com.

5 http://www.vermon.com.

6 http://www.ascension-tech.com.
